# Human Regulatory T Cells From Umbilical Cord Blood Display Increased Repertoire Diversity and Lineage Stability Relative to Adult Peripheral Blood

**DOI:** 10.3389/fimmu.2020.00611

**Published:** 2020-04-15

**Authors:** Keshav Motwani, Leeana D. Peters, Willem H. Vliegen, Ahmed Gomaa El-sayed, Howard R. Seay, M. Cecilia Lopez, Henry V. Baker, Amanda L. Posgai, Maigan A. Brusko, Daniel J. Perry, Rhonda Bacher, Joseph Larkin, Michael J. Haller, Todd M. Brusko

**Affiliations:** ^1^Department of Pathology, Immunology and Laboratory Medicine, Diabetes Institute, College of Medicine, University of Florida, Gainesville, FL, United States; ^2^Department of Molecular Genetics and Microbiology, College of Medicine, University of Florida, Gainesville, FL, United States; ^3^Department of Biostatistics, University of Florida, Gainesville, FL, United States; ^4^Department of Microbiology and Cell Science, University of Florida, Gainesville, FL, United States; ^5^Department of Pediatrics, College of Medicine, University of Florida, Gainesville, FL, United States

**Keywords:** cord blood, peripheral blood, regulatory T cells, Tregs, adoptive cell therapy, scRNA-seq, immunosequencing

## Abstract

The human T lymphocyte compartment is highly dynamic over the course of a lifetime. Of the many changes, perhaps most notable is the transition from a predominantly naïve T cell state at birth to the acquisition of antigen-experienced memory and effector subsets following environmental exposures. These phenotypic changes, including the induction of T cell exhaustion and senescence, have the potential to negatively impact efficacy of adoptive T cell therapies (ACT). When considering ACT with CD4^+^CD25^+^CD127^–/*lo*^ regulatory T cells (Tregs) for the induction of immune tolerance, we previously reported *ex vivo* expanded umbilical cord blood (CB) Tregs remained more naïve, suppressed responder T cells equivalently, and exhibited a more diverse T cell receptor (TCR) repertoire compared to expanded adult peripheral blood (APB) Tregs. Herein, we hypothesized that upon further characterization, we would observe increased lineage heterogeneity and phenotypic diversity in APB Tregs that might negatively impact lineage stability, engraftment capacity, and the potential for Tregs to home to sites of tissue inflammation following ACT. We compared the phenotypic profiles of human Tregs isolated from CB versus the more traditional source, APB. We conducted analysis of fresh and *ex vivo* expanded Treg subsets at both the single cell (scRNA-seq and flow cytometry) and bulk (microarray and cytokine profiling) levels. Single cell transcriptional profiles of pre-expansion APB Tregs highlighted a cluster of cells that showed increased expression of genes associated with effector and pro-inflammatory phenotypes (*CCL5*, *GZMK, CXCR3, LYAR*, and NKG7) with low expression of Treg markers (*FOXP3* and *IKZF2*). CB Tregs were more diverse in TCR repertoire and homogenous in phenotype, and contained fewer effector-like cells in contrast with APB Tregs. Interestingly, expression of canonical Treg markers, such as FOXP3, TIGIT, and IKZF2, were increased in CB CD4^+^CD127^+^ conventional T cells (Tconv) compared to APB Tconv, post-expansion, implying perinatal T cells may adopt a default regulatory program. Collectively, these data identify surface markers (namely CXCR3) that could be depleted to improve purity and stability of APB Tregs, and support the use of expanded CB Tregs as a potentially optimal ACT modality for the treatment of autoimmune and inflammatory diseases.

## Introduction

The human immune system undergoes dramatic changes over the course of a lifetime in order to maintain tissue and organism homeostasis. Highly variable cellular dynamics abound during growth and development in early life. This is particularly apparent during the nascent perinatal period, as the periphery is actively seeded with innate and adaptive immune cells that quickly gain initial exposures to foreign antigens (1). These early priming events must confer protection from pathogens, while also maintaining peripheral tolerance to microbial commensals, inert environmental antigens, and self-tissues. Interestingly, despite inconsistent immune profiles in umbilical cord blood (CB) and newborns, a recent report suggests individuals eventually converge on a common post-natal trajectory for healthy/normal immunological development (1). Efforts to define this common trajectory and the disruptions that give way to immune-mediated diseases represent an essential line of investigation. A growing body of evidence supports the notion that a breakdown in the establishment of peripheral immune tolerance is at the heart of many inflammatory and autoimmune disorders [reviewed in (2)]. While many cell types contribute to immune homeostasis, it is now appreciated that a unique subset of thymic regulatory T cells (tTreg) plays a vital role in establishing and maintaining dominant tolerance to self-antigens in the periphery (3). In fact, tTregs are so essential for maintaining immune homeostasis that loss-of-function mutations in FOXP3, the canonical transcription factor that marks the Treg cell lineage, can result in the lethal multi-organ autoimmune disease referred to as immunodysregulation polyendocrinopathy enteropathy X-linked (IPEX) syndrome [reviewed in (4)].

The identification of tTregs, and the subsequent development of methods for their *ex vivo* isolation and expansion from peripheral blood, has led to an explosion of research interest to harness these cells to control autoimmune diseases, inflammatory disorders, and enable tissue engraftment in the context of transplantation (5–8). The application of *ex vivo* expanded cells to achieve clinical outcomes is broadly referred to as adoptive cell therapy (ACT). ACT with T cells has advanced largely from pioneering work in the cancer immunotherapy space with the goal of tumor-directed immunity (9–15). These endeavors have identified critical factors determining robust clinical response and efficacy. While not comprehensive, these include key parameters of antigen-specificity of the therapeutic T cells (i.e., either polyclonal or antigen-specific) (16–18); lineage stability of the population that is used for ACT (19, 20); and the capacity of the T cells to traffic to proper sites *in vivo*, engraft into tissue microenvironments, and exert their context-dependent effector functions (21, 22). While the desired functions of Tregs in restoring immune regulation contrast those of effector T cells (Teff) targeting cancer, the core concepts governing specificity, stability, and functional capacity are likely to be highly analogous for the use of Tregs to treat autoimmune diseases, including type 1 diabetes (T1D).

Translating these early advances into efficacious therapies with Tregs is likely to require a more robust understanding of Treg biology. Specifically, there is a need for a more complete knowledge of the phenotypic changes that occur over the course of a human lifespan. Murine studies have demonstrated that tTregs generated during the perinatal period display a distinct receptor repertoire and are functionally different from tTregs isolated from mature mice (23). Human adult peripheral blood (APB) Tregs are comprised of a complex mixture of resting and activated subsets (24) and are known to co-opt the transcriptional profiles of the various T helper (T_*H*_) cell subsets they are tasked with suppressing (24–28). In addition, APB Tregs are reported to display lineage instability resulting in effector-like T cell phenotypes (29–31). However, the heterogeneity of Tregs in CB is generally uncharacterized.

Our prior work has demonstrated that tTregs can be isolated from human CB and expanded with exceptional purity and lineage stability (32). Here, we extend our prior studies optimizing Treg expansion protocols to further characterize the transcriptional profile and repertoire characteristics of human Tregs from CB in comparison to those isolated from APB. We employed both bulk transcriptional profiling, as well as single cell RNA sequencing (scRNA-seq) and T cell receptor (TCR) repertoire analyses to characterize CB and APB Treg populations that could be harnessed for ACT. Our novel transcriptional profiling data and repertoire analyses once again reinforce the concept of a phenotypically homogenous and lineage stable Treg population in CB when compared to APB. These studies have implications for identifying optimal cell sources for either autologous or allogeneic ACT applications. Moreover, the scRNA-seq data provide an array of novel cell surface targets that can be leveraged to further optimize Treg isolation strategies for use in Treg ACTs for the induction of immunological tolerance.

## Materials and Methods

### Sample Collection and Processing

**FIGURE 1:**
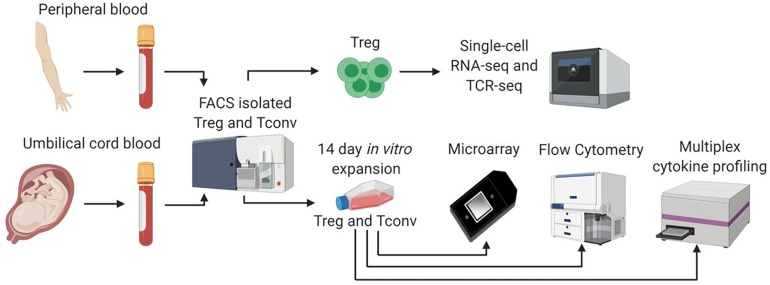
Single cell and bulk sample analysis workflow. We adopted a multifaceted approach to assess differences between CB and APB derived Tregs. Fresh CB Tregs, CB Tconv, APB Tregs, and APB Tconv were fluorescence activated cell sorting (FACS) isolated. Sorted CB Tregs and APB Tregs were directly analyzed by single cell RNA sequencing (scRNA-seq) on the 10x Genomics platform. We assessed single cell gene expression and T cell receptor (TCR) repertoire differences. In addition, freshly sorted CB Tregs, CB Tconv, APB Tregs, and APB Tconv were expanded *in vitro* for 14 days, after which we performed scRNAseq, as well as bulk transcriptional analysis by microarray, flow cytometry and cytokine secretion analysis by Luminex assay.

Fresh CB (processed within 24 h of birth) was obtained from LifeSouth Community Blood Center Corporate Headquarters (Gainesville, FL) into CB units containing 35 mL of citrate phosphate dextrose anticoagulant. CB units (*n* = 7) were delivered to the University of Florida Diabetes Institute (UFDI) and immediately processed for CB mononuclear cells (CBMCs). Leukopaks containing fresh APB(*n* = 6) were purchased from LifeSouth Community Blood Center (Gainesville, FL, United States). These deidentified samples were obtained under an approved IRB exempt protocol at the UFDI. APB samples were processed within 24 h for isolation of peripheral blood mononuclear cells (PBMCs). For CBMC and PBMC isolation, CB and APB samples were subjected to CD4^+^ enrichment with the RosetteSep^®^ Human CD4^+^ T Cell Enrichment Cocktail (STEMCELL Technologies) followed by density gradient centrifugation (Ficoll-Paque PLUS, GE Healthcare) prior to fluorescence-activated cell sorting (FACS). The overall workflow for the experiments reported herein is summarized in Figure 1.

### FACS of CD4^+^ Tregs and Conventional T Cells (Tconv)

CD4^+^ T cell enriched CBMCs and PBMCs were stained with fluorescently labeled antibodies, resuspended at 2 × 10^7^ cells/mL, and sorted on a BD FACS Aria III Cell Sorter (BD Biosciences), as previously described (32). Tregs and Tconv were sorted as CD4^+^CD25^*hi*^CD127^*lo*^ and CD4^+^CD127^+^, respectively.

### T Cell Expansion

Tregs and Tconv from CB and APB were expanded as previously described (32). In brief, sorted Treg and Tconv were incubated with KT64/86 aAPCs at a 1:1 ratio in the presence of exogenous IL-2 and expanded for 14 days with restimulation using anti-CD3 anti-CD28 coated microbeads on day 9 following protocol 1 (32). Expanded CB Tregs, CB Tconv, APB Tregs, and APB Tconv were cryopreserved in CryoStor (Sigma, CS10) and later thawed for batched experiments as described below.

### RNA Extraction and Quality Assessment

Following expansion, 3 × 10^5^ CB Tregs, CB Tconv, APB Tregs, and APB Tconv were lysed in DNA/RNA lysis buffer (Zymo Research) and stored at −80°C. RNA extraction was achieved using ZR-Duet^TM^ DNA/RNA MiniPrep (Zymo Research, Catalog No. D7001), per the manufacturer’s instructions. Quality assessment of RNA was achieved by Experion^TM^ Automated Electrophoresis System (BIO-RAD) using Experion RNA High Sensitivity Reagents and Experion Standard Sensitivity RNA chips following the manufacturer’s protocol. Only samples with a minimum RNA concentration of 10 ng/μL and RNA Quality Index (RQI) ≥9.4 were used.

### scRNA-seq and Library Construction

Gene expression and V(D)J libraries were prepared from 5,000 pre- and post-expansion CB and APB Treg cells using the Chromium Single Cell 5′ Bead and Library Kit v1 and the Chromium Single Cell V(D)J Human TCR Analysis Kit (10X Genomics). Libraries were sequenced on an Illumina HiSeq instrument at a target read depth of 50,000 reads per cell.

### Processing of Sequencing Reads and Generation of Gene-Barcode Matrices

Raw sequencing reads were processed using Cell Ranger v3.0.0 to create a raw (unfiltered) gene-barcode matrix. Briefly, Cell Ranger mkfastq was used to make fastq files from bcl files. Next, Cell Ranger count was used for aligning sequencing reads to the hg19 reference genome (refdata-cellranger-hg19-3.0.0), obtained from^1^ using STAR (33). For confidently mapped reads (as defined by Cell Ranger), Unique Molecular Identifier (UMI) sequences were collapsed and the number of UMI reads per gene were stored in the raw gene-barcode matrix^2^.

### Filtering of Barcodes/Quality Control and Normalization

Quality control was performed for scRNA-seq data from pre- and post-expansion APB and CB Tregs (Supplementary Figure S1). Barcodes associated with droplets containing cells were distinguished from ambient RNA droplets using the emptyDrops algorithm implemented in the DropletUtils R/Bioconductor package (34). Briefly, each barcode is tested for deviations from the estimated ambient RNA profile as defined by barcodes with 100 UMIs or less. Barcodes with a false discovery rate adjusted *p*-value < 0.01 were retained after this initial filter. A second filter based on the inflection point in the UMI rank versus total UMI curve was used for more stringent identification of cellular barcodes (Supplementary Figure S1A). Next, we filtered on commonly used quality control measures, such as the total number of UMIs per cell (library size), the number of genes expressed, and the percentage of mitochondrial reads per cell to identify cells with low RNA content, possible doublets, and presumably dead or damaged cells. Cells with a total UMI count or number of genes expressed greater than or less than three median absolute deviations (MADs) from the median were removed. Additionally, cells with a percentage of mitochondrial reads greater than three MADs from the median were removed. This filtering was implemented using the isOutlier function in the scran R/Bioconductor package (35) (Supplementary Figures S1B,C). Additionally, cells with more than one unique *TRB* chain and two unique *TRA* chains as defined by the concatenated V-gene, complementarity determining region 3 (*CDR3*) sequence, and J-gene were excluded as presumed doublets (Supplementary Figure S1D). 4320 cells from the APB and 4842 cells for the CB pre-expansion samples, and 4403 cells from post-expansion APB and 3842 cells from post-expansion CB Treg passed these filters and were used in downstream analyses. To remove variation in the number of molecules detected per cell, residuals from regularized negative binomial regression with library size as a covariate was used as described in (36) and implemented in the SCTransform function in Seurat v3.1 (37). Briefly, a negative binomial regression model is fit for each gene with the number of molecules per cell as a covariate and the read-count of the cell as the dependent variable. This method selects stable model parameters that are robust to overfitting by pooling parameter estimates across genes with similar abundances.

### Dataset Integration and Dimensionality Reduction

The cord blood and adult peripheral blood datasets were integrated as detailed in (38) and implemented in Seurat. Briefly, canonical correlation analysis (CCA) was performed to identify shared sources of variation across the datasets, and mutual nearest neighbors in the CCA space were identified to produce anchors between datasets. For pre-expansion datasets, highly variable genes accounting for the majority of the heterogeneity within each sample were identified by ranking genes based on variance of the residuals from the regularized negative binomial regression model described above, again as described in (36) and implemented in the SCTransform function in the Seurat R package (37, 39). For post-expansion datasets, the corresponding variable features from the pre-expansion state were used, as the variable features post-expansion were dominated by cell-cycle driven expansion-related genes that were not of primary interest. Using these features, anchors between the datasets which correspond to similar cells across datasets were identified using the FindIntegrationAnchors function, and this was used as input into the IntegrateData function to generate an integrated dataset. For dimensionality reduction, expression values for each gene in the integrated dataset were scaled to have a mean of zero and standard deviation of one using the ScaleData function, and principal component analysis (PCA) was run on this matrix using the RunPCA function in Seurat (37, 39). For visualization, Uniform Manifold Approximation and Projection (UMAP), a common dimensionality reduction method in scRNA-seq, plots were created based on the top 20 principal components using the RunUMAP function in Seurat.

### Clustering and Cluster Differential Expression Analysis

Cells were clustered into groups of similar transcriptomic profiles using graph-based clustering on the first 20 principal components of the integrated dataset. Briefly, a shared nearest neighbors graph was created based on the Jaccard similarity of the sets of the 20-nearest neighbors for each cell, as implemented in FindNeighbors function in Seurat (37, 39). Clusters were then identified by partitioning this graph using the Louvain community detection algorithm with a resolution of 0.4, as implemented in the FindClusters function in Seurat (37). Clusters sizes and the relationship between clusters at different resolutions were analyzed to determine this value (Supplementary Figures S5, S6) (40). DE genes across clusters were identified by comparing each individual cluster with the remaining pooled clusters for each sample using the Wilcoxon rank sum test implemented in the wilcoxauc function in the presto R package (41). *P*-values for each cluster from each sample were then combined using Wilkinson’s method as implemented in the minimump function in the metap package (42) to identify conserved markers across datasets.

### TCR Clonotype Assignment and Evenness Profile Calculation

Clonotypes were assigned to cells based on unique paired *TRA-TRB* V-gene/CDR3/J-gene sequences. Only cells with one β-chain and one α-chain were assigned clonotypes to prevent artificial inflation of clone counts due to reduced information about the sequence. Evenness profiles were calculated as initially described (43). Briefly, for sample-level analysis, clonotypes were tabulated, and frequency vectors for each clonotype within a sample were calculated. Evenness profiles based on the exponential of Hill diversity were computed for α in the range 0–10, with step size 0.2, where Eα=DαDα=1 and ${}^{\alpha }D\left(\text{f}\right)={\left(\sum_{i=1}^{\text{n}}f_{\text{i}}^{\alpha }\right)}^{\frac{1}{1-\alpha }}$, where ^α^*E* is the evenness for a given α and ^α^*D* is the Hill diversity for a given α, and *f* is the frequency vector. When α = 1, while Hill diversity is not defined, it tends to Shannon entropy (43). This resulted in a 51-dimensional evenness profile for each sample. A large range of α was used to capture differences in clonal expansion across the clonal frequency distribution, as the majority of the clonotypes were single occurrence, and an increased α results in higher frequency clones being given a greater weight. The same procedure was followed for the cluster-level analysis, except each cluster from each sample is treated as an independent sample. Overall, the evenness profile is a low-dimensional vector containing the majority of the information contained in a clonal frequency distribution (44).

### Microarray Studies

Post-expansion Treg transcript analysis was performed as previously described (45). Briefly, mRNA was reverse transcribed and amplified. Resulting cDNA was fragmented and labeled using the GeneChip WT Plus Kit and subsequently, hybridized onto the Clariom S Human Array (Thermo Scientific), following the manufacturer’s procedures. Arrays were scanned with the GeneChip Scanner 3000 7G using AGCC software and subsequently normalized using RMA as implemented in Partek 6.6. GEO Accession #: GSE137301.

### Differential Expression Analysis

The R/Bioconductor package limma (46) was used for differential expression of genes using a linear model using the lmFit function with a model matrix with no intercept and fixed effect for treatment (e.g., CB Treg, APB Treg, CB Tconv, and APB Tconv), blocking on donor, and specifying an inter-donor correlation using duplicateCorrelation, effectively treating donor as a random effect. Contrasts were specified using makeContrasts, and the contrasts were fit using contrasts.fit. Moderated t-statistics were then computed using the empirical Bayes moderation as implemented in the eBayes function.

### Absolute Telomere Length Assay

APB and CB Treg DNA was assayed using the Absolute Human Telomere Length Quantification qPCR Assay Kit (ScienCell) according to the manufacturer’s instructions, with the exception of the qPCR master mix, for which we used Syber Select Master Mix (Applied Biosystems). Briefly, DNA were isolated using the DNEasy Blood and Tissue Kit (Qiagen), quantified using a Qubit Fluorometer (Thermo Fisher), after which 5 ng was input into the assay per subject. Data were acquired on a Roche LightCycler480 instrument, exported into Excel and analyzed in GraphPad PRISM v8.

### Flow Cytometry

Expanded cryopreserved Tregs and Tconv from CB and APB were thawed in RPMI complete media and stimulated with phorbol myristate acetate (PMA; 10 ng/mL) and ionomycin (500 nM) for 4 h at 37°C with the addition of Golgistop (BD Biosciences; 0.66 μl/mL). Cells were stained for surface and intracellular markers to assess differentiation and effector markers, chemokine receptors and activation status (Supplementary Table S1). Data were collected on an LSRFortessa (BD Biosciences) and analyzed using FlowJo software (Tree Star, Inc). For each marker, the percentage of cells positive for the marker was modeled with a mixed effects model using the lmer function in the lme4 package (47) with treatment (e.g., CB Treg, APB Treg, CB Tconv, and APB Tconv) as a fixed effect and donor as a random effect. Pairwise contrasts for treatment were computed using the emmeans function in the emmeans package (48). For supplementary experiments, expanded cryopreserved APB and CB Tregs or CBMC and PBMC were thawed in RPMI complete media and restimulated with αCD3/28-coated microbeads at a 1:1 ratio (milltenyi) or soluble αCD3 (2 μg/mL, clone HIT3a, BD Biosciences) and αCD28 (1 μg/mL, clone 28.2, BD Biosciences), respectively, for 48 h at 37°C with the addition of Golgistop (BD Biosciences; 0.66 μl/mL) for the last 4 h. Data were collected on a Cytek Aurora and analyzed as above with statistics computed and data plotted using Graphpad PRISM software v8, as indicated in figure legends.

### Multiplexed Cytokine Detection

Isolated expanded CB Tregs, CB Tconv, APB Tregs, and APB Tconv were stimulated in a 96-well plate with PMA (10 ng/mL) and ionomycin (500 nM) for 4 h at 37°C. IL-2, IL-10, IL-12 (p40), IL-12 (p70), IL-19, IL-20, IL-22, IL-26, IL-27 (p28), IL-28A, IL-29, and IL-35 were detected in the supernatant using the Bio-Plex Pro Human Treg Cytokine 12-Plex Panel (Bio-RadR) according to the manufacturer’s procedures with the following modification. Standards were diluted in standard Diluent HB as opposed to culture medium to generate a seven-point curve. For each cytokine, log10 (concentration) was modeled with a mixed effects model using the lmer function in the lme4 package (47) with treatment (e.g., CB Treg, APB Treg, CB Tconv, and APB Tconv) as a fixed effect and donor as a random effect. Pairwise contrasts for treatment were computed using the emmeans function in the emmeans package (48).

### Data Visualization

Data were visualized using the following R packages: ggplot2 (49), ComplexHeatmap (50), scanalysis (51), ggexp (52), and clustree (40). Flow cytometry data were analyzed in FlowJo software (Tree Star, Inc.) and raw data were exported to GraphPad PRISM v8 or R for statistical analysis.

### Code Availability

An R package with runner scripts to reproduce all analyses and figures in this manuscript are available at https://github.com/keshav-motwani/tregPaper (53).

## Results

### scRNA-seq Identifies Contaminants in Pre-expanded Tregs

**FIGURE 2:**
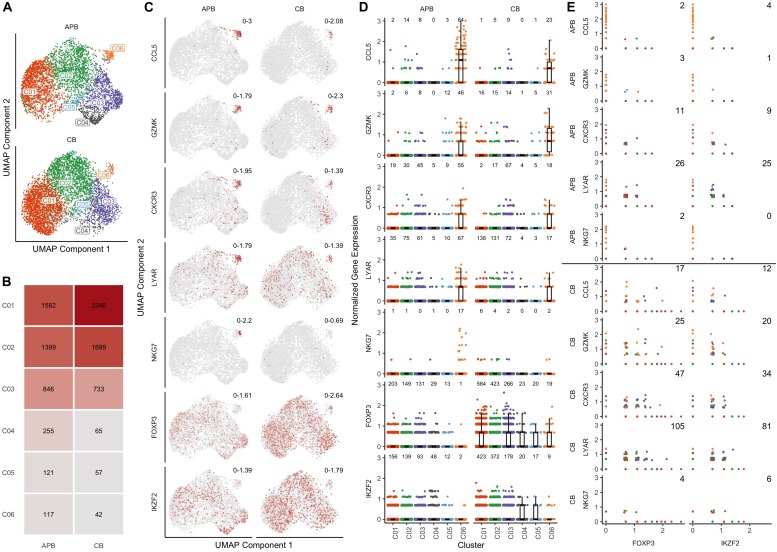
Single cell gene expression profiles of unexpanded APB Tregs and unexpanded CB Tregs show the presence of a T_*H*_1/T_*H*_17-like contaminant cluster in APB. **(A)** UMAP plots for APB Tregs (*n* = 1, top) and CB Tregs (*n* = 1, bottom) colored by the assigned cluster number (C1-C6) shows the presence of a subpopulation (C06) that is not along the main trajectory and appears more prevalent in APB. **(B)** Absolute number of cells belonging to each cluster and relative abundances for each cluster within unexpanded APB Tregs (left) and unexpanded CB Tregs (right) show differences in cluster composition. Notably, C06 was comprised of 117 cells (3%) of APB Tregs versus 42 cells (1%) of CB Tregs. **(C)** For APB Tregs (left) and CB Tregs (right), UMAP plots are colored by expression of the five most differentially expressed genes in C06 (*CCL5*, *GZMK*, *CXCR3*, *LYAR*, and *NKG7*) as well as two canonical Treg genes (*FOXP3* and *IKZF2*). Numbers in the top right of each plot indicate the minimum and maximum expression for that sample. Points are colored based on the expression of the gene, with gray being the lowest value and dark red the highest value for that feature across both samples. **(D)** Visualizing expression of *CCL5*, *GZMK*, *CXCR3*, *LYAR*, *NKG7*, *FOXP3*, and *IKZF2* shows increases in “contaminant” gene expression in C06 and decreases in canonical Treg gene expression within this cluster from APB Tregs (left) and CB Tregs (right). The number of cells with nonzero expression of each feature in each cluster is annotated at the top of each plot. **(E)** For all clusters, pairwise scatterplots between canonical Treg genes *FOXP3* (left) and *IKZF2* (right) and the identified “contaminant” genes (*CCL5*, *GZMK*, *CXCR3*, *LYAR*, and *NKG7*) shows increased co-expression in CB Tregs (lower) compared to APB Tregs (upper). The annotated number represents the number of cells with nonzero expression for both genes. **(D,E)** For each data point, the color corresponds to the cluster number as presented in **(A)**.

We sought to identify differences in the composition of native (i.e., unexpanded) CB and APB Tregs at the single cell level that might contribute to non-Treg contaminants in a post-expansion cell product for use in ACT. After identification of 4842 high quality cells in CB (*n* = 1 subject) and 4320 in APB (*n* = 1 subject), datasets were normalized for cell-specific biases related to sequencing depth using the residuals of regularized negative binomial regression as described in (36). To enable direct comparisons between APB and CB, the datasets were integrated by identifying anchors between similar cells across datasets (38). We performed graph-based clustering on the top 20 principal components (PCs) of the integrated data, identifying a total of 6 clusters which are overlaid on a reduced dimensional representation of the first 20 PCs using UMAP (54) (Figure 2A). From visual inspection of the first two UMAP components (i.e., UMAP1 and UMAP2; Figure 2A), cells in clusters C01-C05 are largely clumped together, but cluster C06 is more of an outlier (Figure 2A), and it is more pronounced in the APB sample (∼3% of cells in APB versus ∼1% in CB) (Figure 2B). To understand the underlying biology in each of the 6 subpopulations, we computed differentially expressed genes between each cluster and the rest of the cells in the dataset. For C06 in particular, the top five differentially expressed (DE) genes (ranked on *p*-value) were *CCL5*, *GZMK, CXCR3, LYAR*, and *NKG7* (Supplementary Table S2), as shown in the UMAP plots colored by relative expression (Figure 2C). *CXCR3*, *CCL5*, and *NKG7* have all been associated previously with T_*H*_1 migratory capacity (55, 56) and phenotype (57), while the expression of *GZMK* and *LYAR* likely indicate a cytotoxic and activated population (58, 59). Figure 2C also depicts the canonical Treg markers, *FOXP3* and *IKZF2*, which were highly expressed in the majority of APB and CB Treg clusters but only lowly expressed in C06. Figure 2D shows the expression of these seven genes across all clusters where notably, there is a decrease in *FOXP3* and *IKZF2* expression and an increase in the C06 DE genes (*CCL5*, *GZMK, CXCR3, LYAR*, and *NKG7*). These trends are much more prominent in APB as compared to CB, due to the greater number of contaminant cells in APB. Moreover, there is greater co-expression of *FOXP3* and *IKZF2* with these five “contaminant cell” genes in CB compared to APB (Figure 2E). This could potentially indicate that contaminants expressing these T_*H*_1-associated genes are present in both CB and APB, but the T_*H*_1-like contaminants in CB still retain a Treg phenotype while in APB, they lose their regulatory phenotype and adopt an effector-like program. Further examination of the top 50 DE genes in C6 (Supplementary Table S2, ranked on combined *p*-value) shows upregulation of additional T_*H*_1-associated genes including *BHLHE40* (60), *IFNG* (61), and *TBX21* (62); T_*H*_17-related genes including *KLRB1*, which encodes CD161 (63), and *TGFB1* (64); as well as *IL12RB2* (65), shown to be expressed highly in T_*H*_1/17 cells, which collectively suggests this contaminant population to belong to the recently characterized T_*H*_1/17 subset (66). These data indicate that the CB Treg transcriptomic profile is more homogenous as a lineage as compared to APB Tregs, which contain non-Treg T_*H*_1/17 contaminants with cytotoxic and pro-inflammatory potential.

### CB Treg Repertoire Is Highly Diverse and Enriched in TCRs Associated With Self-Reactivity

**FIGURE 3:**
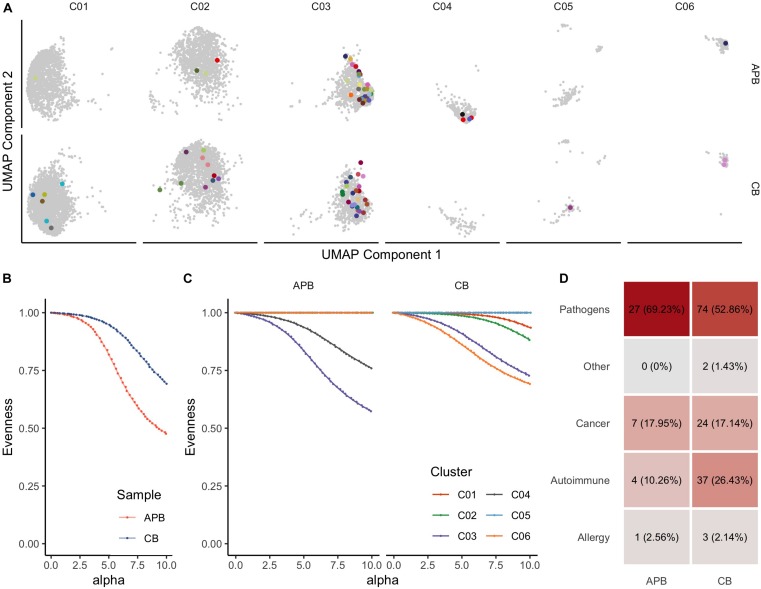
T cell receptor (TCR) profiling from scRNA-seq data identifies clonally expanded subpopulations in pre-expanded Tregs. **(A)** UMAP plot colored by clonotypes with single occurrences (gray) and multiple occurrences (each clonotype distinctly colored) identifies clusters with expansion (UMAP Components 1 and 2 are the same as those presented in Figure 2). **(B)** Alpha value (i.e., clonotype frequency) versus evenness value (43) for APB Tregs (orange) and CB Tregs (blue) shows that along the range of the frequency distribution, APB Tregs show decreased evenness at all alpha values, thus exhibiting greater clonal expansion than CB Tregs. **(C)** When APB Tregs (*n* = 1, left) and CB Tregs (*n* = 1, right) are split by cluster (C01-C06, as defined in Figure 2), APB C03 and APB C04 contain expanded subpopulations, while CB C01, C02, C03, and C06 are also moderately expanded. **(D)** Putative antigen specificity composition of TCRs in each sample show an increased number of clones mapping to known reactivities, including autoimmune clones, in CB Tregs (right) compared to APB Tregs (left).

Immune tolerance is initiated by tTregs that seed the periphery in early life, as reviewed previously (1, 23, 67). We and others have shown from bulk *TRB* immunosequencing that Tregs generally express a diverse repertoire of TCRs (32, 68–71). Here, we extended these studies to include paired *TRA* and *TRB* receptor analysis in unexpanded APB and CB Tregs. To understand how clonal expansion of cells related to their phenotype as represented on the UMAP plots, we compared expanded CDR3 sequences spanning the V and J genes for TCR-α and TCR-β chains (i.e., *TRAV*, *TRAJ*, *TRBV*, *TRBJ*) wherein every clone with a single occurrence was presented in gray while expanded clones with two or more occurrences were assigned a unique color (Figure 3A). Clonal expansion was observed in clusters 1, 2, 3, 4 (C01, C02, C03, and C04, respectively), and C06. To further characterize clonal expansion in each sample and the extent within each cluster we assessed receptor evenness profiles, which reflect the frequency vectors’ distance from a uniform distribution and serve as a normalized diversity metric (Figure 3B) (43, 44). Overall, APB Tregs showed reduced diversity compared to CB Tregs (Figure 3B). This likely reflects TCR enrichments over time, presumably from chronic antigen exposures and selective pressures in the periphery. To determine if these differences were due to the influence of specific clusters, we compared the receptor evenness of each cluster between APB and CB (Figure 3C). Clusters 1 (C01) and 2 (C02) were moderately expanded in CB alone (Figure 3C, **red** and **green**), and were found to express genes related to Treg development, stability, and migratory capacity [*JUNB* (72), *DUSP2* (73), and *ITGB1* (74)], while the latter expressed genes associated with Treg activation and suppressive function [*IDI1* (75), *FCRL3* (76), and *ID3* (77)]. APB Treg cluster 3 (C03) demonstrated reduced receptor evenness in both APB and CB (Figure 3C, purple), and expressed genes related to T cell activation and memory phenotypes, namely *S100A4*, *S100A10*, *DUSP4*, *LGALS1*, and *LGALS3* (Supplementary Table S2) (78–81), as well as class II HLA and co-stimulatory molecules *TNFRSF4* and *TNFRSF18* (82, 83) and *PRDM1* (BLIMP-1) (Supplementary Table S2), likely representing a population of activated memory Tregs (84). C04 was also expanded in APB alone (Figure 3C, brown), and possessed an eTreg phenotype, with expression of *CCR4* (86) (Supplementary Table S2). Lastly, C06 (Figure 3C, orange), which is discussed extensively above, was expanded in CB, though the co-expression of T_*H*_1-associated genes with *FOXP3* and *IKZF2*, which encodes Helios (Figure 2), suggests this population to potentially represent differentiated Tregs capable of suppressing the T_*H*_1 effector lineage, as opposed to a T_*H*_1 contaminant (87). Hence, among clusters exhibiting clonal expansion, the majority appeared to retain a regulatory identity with the exception of C06 within APB. Moreover, we were able to identify a cluster with a similar phenotype to C06 within expanded CB and APB Treg (C08, Supplementary Figure S2, and Supplementary Table S3), which expressed *KLRB1* and *IFNG*, as well as *CD40LG* (88), cytotoxic molecules *GZMA* and *GZMB* (58), and additional T_*H*_17-associated genes *CCR6* (89) and *RORC* (90), indicating that the pre-expansion C06 cluster phenotype may still be relevant after expansion. Notably, we found post-expansion CB Treg to exhibit increased receptor evenness as compared to APB Treg (Supplementary Figure S3). Overlap between pre- and post-expansion contaminant cluster gene signatures is shown in 1Supplementary Figure S4. Additionally, both pre- and post-expansion, the contaminant cluster was a distinct cluster that was robust to changes in cluster resolution (Supplementary Figures S5, S6). Lastly, to understand the reactivities of the Tregs in APB and CB, we defined CDR3β sequence specificities using the manually curated catalog of pathology-associated T cell receptor sequences (McPAS-TCR) (91), matching the observed sequence composition (CDR3B) to sequences associated with putatively annotated reactivities and pathological conditions. Interestingly, we observed a greater total number of CB Treg sequences corresponding to known predicted targets as compared to APB Tregs, and though there are comparable distributions of predicted reactivities, we observed an increased number of sequences with autoreactive specificity in CB Tregs (Figure 3D and Supplementary Figure S4D). These data suggest that even in the polyclonal state, CB Treg may be optimal for broad tissue engraftment with more clones expressing TCRs reactive to autoantigens when compared to Tregs derived from APB.

### Expanded CB Treg Retain Lineage Stability and Phenotype

**FIGURE 4:**
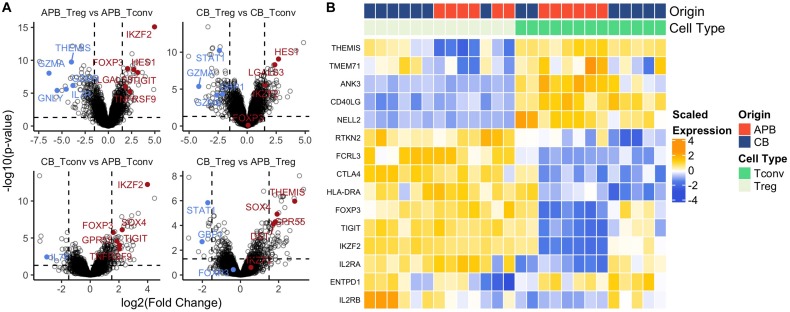
Bulk gene-expression profiles from expanded cells show increased regulatory phenotype in both Tregs and Tconv from CB compared to APB. **(A)** Volcano plots showing log2 (Fold Change) versus -log10 (adjusted *p*-value) annotated with differentially expressed genes (155) colored by downregulation (blue) and upregulation (red). **(B)** Heatmap of canonical Treg and Tconv genes shows clustering by cell type (Treg (*n* = 13) versus Tconv (*n* = 13) and also sample origin (APB (*n* = 14) versus CB (*n* = 12).

Achieving clinically effective Treg numbers in ACT often requires cell expansion (92). Moreover, it is essential that Tregs maintain a regulatory identity and the capacity for cycling and activation post-expansion. We previously determined CB Treg to maintain a naïve phenotype post-expansion (32) which is substantiated by our observation of increased telomere length in expanded CB Treg (CB mean: 383.4 ± 16 kb, APB mean: 258.2 ± 11 kb, Supplementary Figure S7). However, there is a paucity of data examining transcriptomic differences between expanded CB and APB Tregs. Therefore, we sought to address this by characterizing the transcriptome of CB and APB derived Tregs and Tconv by microarray after a 14 day *in vitro* expansion period. As expected (32, 93), among the top 30 DE genes between APB Treg and APB Tconv were the canonical Treg transcription factors *FOXP3* and *IKZF2*, along with various negative regulators and functional molecules, namely *TIGIT* and *TNFRSF9* (88), while APB Tconv preferentially expressed pro-inflammatory and cytotoxic mediators such as *GZMA*, *IL-7R*, *GZMB*, *GNLY*, and *IL18RAP* (Figure 4A and Supplementary Table S4). Differences in expression of canonical Treg genes were less robust between CB Tconv and CB Tregs (Figure 4A and Supplementary Table S4). Nevertheless, in CB Treg versus CB Tconv, we observed higher expression of *LGALS3* and *LGMN*, which enhance *FOXP3* expression (94), as well as *HES1*, which promotes TGF-β signaling (95) (Figure 4A and Supplementary Table S4). Moreover, in CB Tconv versus APB Tconv, we observed increased *IKZF2*, *TIGIT*, *TNFRSF9* and *SOX4*, the latter of which is induced by TGF-β signaling (96) (Figure 4A and Supplementary Table S4), supporting the notion that CB Tconv may adopt a more regulatory phenotype than APB Tconv. Interestingly, relative to CB Tregs, APB Tregs expressed higher levels of *GBP1* and *STAT1* (Figure 4A and Supplementary Table S4), previously shown to be involved in IFN-γ signaling (97) and to serve as a driver of T_*H*_1 differentiation (98), respectively. In addition, compared to APB Tregs, CB Tregs were enriched for markers promoting homing to the gut [*GPR55* (99)], adhesion and migration through the basal lamina [*DST* (100)], and stem-cell and recent thymic emigrant phenotypes [*TCF4* (101) and *THEMIS* (102)] (Figure 4A and Supplementary Table S4). To summarize these data, heatmaps of a selection of differentially expressed genes between Treg and Tconv show that both CB and APB Tregs highly expressed canonical Treg genes (e.g., *FOXP3*, *IKZF2*, *CTLA4*, and *TIGIT*), while APB Tconv largely lacked expression of these genes (Figure 4B). Interestingly, CB Tconv expressed some markers typically attributed to a Treg phenotype, namely *TIGIT*, *IKZF2*, and *FOXP3* (Figure 4B), again suggesting a more immunoregulatory phenotype than their APB Tconv counterparts or the selective expansion of Treg following the initial cell isolation. Hence, bulk transcriptomic profiling of CB Tregs supports their lineage stability and retention of a suppressive phenotype post-expansion.

### Expanded CB Tregs Exhibit a Highly Activated and Suppressive Phenotype

**FIGURE 5:**
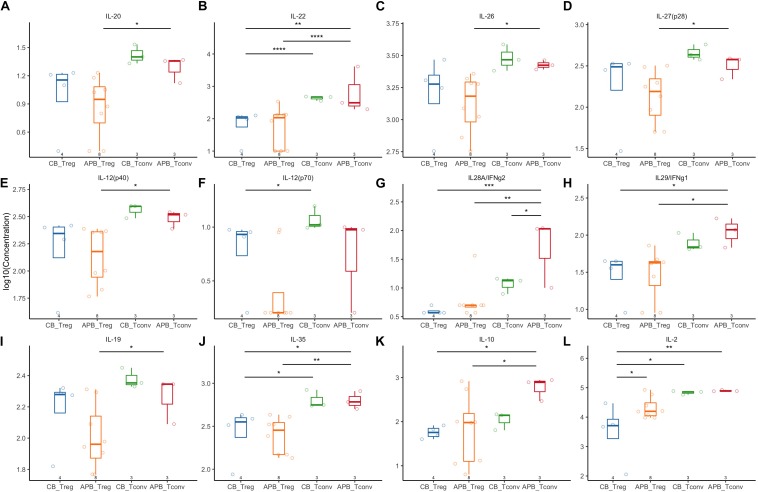
Expanded APB Tregs and CB Tregs have similar cytokine production profiles. Expanded CB Tregs (blue, *n* = 4), APB Tregs (orange, *n* = 8), CB Tconv (green, *n* = 3), and APB Tconv (red, *n* = 3) were activated with PMA/ionomycin and cytokine production detected by Luminex assay. CB Tregs and APB Tregs displayed limited differences in cytokine production profile, as there were no differences in production of **(A)** IL-20, **(B)** IL-22, **(C)** IL-26, **(D)** IL-27, **(E)** IL-12p40, **(F)** IL-12p70, **(G)** IL-28, or **(H)** IL-29. APB and CB Tregs produce similar amounts of immunosuppressive cytokines **(I)** IL-19, **(J)** IL-35, and **(K)** IL-10, while APB Tregs produced significantly more **(L)** IL-2 relative to CB Tregs. When comparing CB Tconv versus APB Tconv, **(A–J,L)** cytokine production was similar except APB Tconv produced significantly more **(K)** IL28A/IFNg2 as compared to CB Tconv. Data were analyzed in R with a mixed effects model using the lmer function in the lme4 package, as noted in Materials and Methods. *p < 0.05, **p < 0.01, ***p < 0.001, ****p < 0.0001.

**FIGURE 6:**
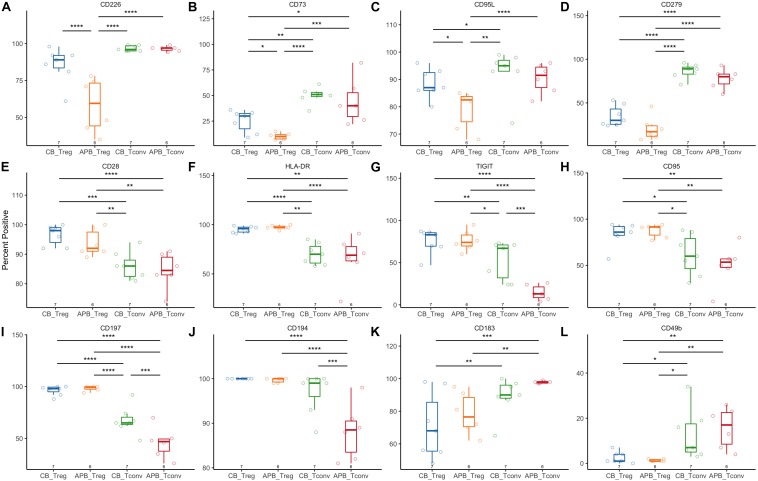
Flow cytometric analysis shows increased activation and suppressive phenotype of CB Tregs compared to APB Tregs. CB Tregs and CB Tconv (*n* = 7), and APB Tregs and APB Tconv (*n* = 6) were assessed for differences in phenotype post-expansion. Frequencies of **(A)** CD226, **(B)** CD73, and **(C)** CD95L positive cells were increased in CB Tregs versus APB Tregs. CB Tregs and APB Tregs possessed similar frequencies of **(D)** CD279^+^ (PD-1^+^) cells, while CD279^+^ cells were more prevalent among CB Tconv and APB Tconv. CB Tregs and APB Tregs also possessed similar proportions of cells positive for **(E)** CD28, **(F)** HLA-DR, **(G)** TIGIT, **(H)** CD95, **(I)** CD197 and **(J)** CD194, with Tregs overall displaying an increased proportion of cells positive for these as compared to Tconv. CB Tconv and APB Tconv displayed an increased proportion of cells expressing **(K)** CD183 (CXCR3) and **(L)** CD49b as compared to Tregs. Data were analyzed in R with a mixed effects model using the lmer function in the lme4 package, as noted in Materials and Methods. *p < 0.05, **p < 0.01, ***p < 0.001, ****p < 0.0001.

Next, we expanded CB and APB derived Tregs and Tconv, restimulated with PMA/ionomycin, and examined their phenotype and cytokine production by flow cytometry and Luminex assay, respectively. We found cytokine production and phenotypic profiles to be mostly similar for expanded CB Tregs versus APB Tregs (Figure 5 and Figure 6, blue and orange) and for expanded CB Tconv versus APB Tconv (Figure 5 and Figure 6, **green** and **red**) with the most dramatic differences observed between Tregs and Tconv, regardless of the source (CB or APB). Expectedly, CB and APB Treg produced limited pro-inflammatory and effector cytokines (Figures 5A–H) relative to Tconv (103–108), and though we observed no differences in the production of immunosuppressive or effector Treg-associated cytokines (109, 110) between the two subsets (Figures 5I–K), we did observe APB Treg to produce increased IL-2 relative to CB Treg (Figure 5L). This could be indicative of non-Treg contaminants, consistent with known Treg reliance on exogenous IL-2 (111). Phenotypically, we found CB Tregs to be more activated than APB Treg, as evidenced by an increased frequency of CB Tregs expressing the costimulatory molecule CD226 (Figure 6A). Moreover, CB Treg possessed increased proportions of cells expressing CD73 and CD95L (Fas Ligand (FasL) (Figures 6B,C), while CD279 (PD-1) was not significantly different between APB and CB Tregs (Figure 6D). This suggests CB Treg to have increased capacity for functional suppression via conversion of extracellular ATP to adenosine [CD73 (112)] and activation induced cell death [AICD; FasL (113)], without succumbing to Treg exhaustion [PD-1 (114)] (Supplementary Figure S8). CD28^+^ cells were more frequent among CB Tregs and APB Tregs as compared to CB Tconv and APB Tconv (Figure 6E); this was not surprising given that CD28 signaling is essential for Treg development (115), promotes lineage stability and anti-inflammatory cytokine production (116). CB Tregs and APB Tregs both displayed an increased frequency of cells expressing the activation marker HLA-DR as compared to APB Tconv and CB Tconv (Figure 6F). Moreover, compared to APB Tconv, CB Tregs, APB Tregs and CB Tconv displayed increased percentages of cells expressing the suppressive and activation marker, TIGIT (Figure 6G), in agreement with our post-expansion bulk sequencing data (Figure 4). Compared to APB Tconv, Tregs derived from either APB or CB were enriched for cells expressing CD95, a memory Treg marker (Figure 6H) (117). CB Tregs, APB Tregs, and CB Tconv also contained a greater percentage of cells positive for the chemokine receptors CD197 and CD194 as compared to APB Tconv (Figures 6I,J), potentially reflective of increased homing potential to secondary lymphoid organs and the skin, respectively (118, 119). Importantly, compared to Tconv, CB and APB Treg possess a reduced percentage of cells expressing CD183 (CXCR3), a T_*H*_1-related chemokine receptor (Figure 6K). Moreover, we found CB Treg to possess fewer FOXP3^+^HELIOS^–^ cells pre- and post-expansion, with an increase in CXCR3 (gMFI and percent positive) noted on this subset within APB (Supplementary Figures S9, S10). Finally, we assessed CD49b expression as a marker of T_*r*_1 cells, which have been shown to exhibit similarity to T_*H*_1 cells and to express CXCR3 (120). T_*r*_1 differentiation has been reported to occur in the presence of pro-inflammatory cytokines (121, 122), and while these cells have the potential to be potent suppressors, they lose this capability in the absence of IL-10 while still retaining a cytotoxic program (123), thus making their inclusion in an ACT product a potential risk. The frequencies of CD49b^+^ cells were low and did not differ between CB Tregs versus APB Tregs (Figure 6L), indicating a lack of T_*r*_1 differentiation (124). Cumulatively, these data when considered in addition to the transcriptional profiles suggest that CB Tregs retained a highly activated status, suppressive phenotype, and distinct homing capacity when compared APB Tregs.

## Discussion

Tregs, when used in the context of ACT, are expected to function as “living drugs” and exert their suppressive functions via numerous mechanisms including expression of negative surface regulators, IL-2 sequestration, and the production of immunoregulatory cytokines as well as other suppressive mediators (125). Importantly, these cells have the potential to traffic to relevant sites of inflammation, and are capable of bystander suppression and infectious tolerance (126). It is precisely this combination of therapeutic properties that has generated great interest in harnessing Tregs for the establishment of long-term tolerance in situations of autoimmunity and/or transplantation. In fact, many of these concepts have been repeatedly demonstrated in animal models of disease but have not, to date, been broadly translated into effective therapies in humans. We would speculate that translation is hampered both by practical considerations (e.g., cost and feasibility of large-scale production) as well as incomplete knowledge of the optimal cellular properties needed to maximize Treg specificity and function in humans.

We previously demonstrated the potential to isolate and expand previously cryopreserved CB-derived Tregs with increased lineage stability relative to APB (32). Given that it is now possible to store CB to create large population biobanks, it is now feasible to consider options for both autologous and HLA-matched allogeneic CB sourcing for ACT applications. Additionally, the utilization of banked CB units avoids the necessity for large blood draws or leukapheresis procedures to obtain sufficient Treg quantities for expansion. This would be highly desirable in pediatric and autoimmune subjects, many of whom may exhibit lymphopenia, increased inflammatory cell populations as potential contaminants, or express multiple genetic susceptibility alleles that may negatively affect Treg function (127–129).

In an effort to better understand how CB compare to APB Tregs, we conducted scRNA-seq of pre- and post-expansion Tregs (Figure 1). This analysis demonstrated an enriched cell cluster (herein referred to as C06) in APB defined by a gene expression profile associated with the T_*H*_1 (*CCL5*, *CXCR3*, *BHLHE40*, *NKG7*, *IFNG*, *TBX21*) and T_*H*_17 (*KLRB1*, *TGFB1, IL12RB2, CCR6, RORC*) lineages, as well as activation and cytotoxicity (*GZMK*, *LYAR*), indicating this population may belong to the recently characterized T_*H*_1/17 lineage (66). Interestingly, functional Treg markers (*FOXP3*, *IKZF2*) were downregulated in this cluster amongst APB but not CB Tregs, supporting the notion that CB Tregs are a more homogenous population with increased lineage stability and reduced Teff contaminants. Enrichment of cells expressing pro-inflammatory genes, namely *IFNG*, could have negative implications for Treg plasticity and function. Indeed, IFNγ^+^ Tregs have been documented in healthy donors but are enriched in various autoimmune conditions including multiple sclerosis (130) and T1D (31), and have impaired suppressive capacity (131). Moreover, CCL5 and CXCR3 have been shown to be induced following IFNγ signaling (132, 133) and to influence the trafficking of GvHD-promoting proinflammatory T cells (134, 135); hence, we propose that the cellular phenotype of C06 present in APB Treg should be further investigated for its potential to negatively impact the success of Treg ACT.

An important consideration in the development of Treg therapies is antigen specificity, as autoreactive thymocytes with lower to moderate affinity TCRs are thought to preferentially differentiate to the Treg lineage in an autoimmune regulator (AIRE)-dependent manner (136). This bias toward self-reactivity represents a critical paradigm in the suppression of autoreactive Teff in the periphery. However, achieving antigen-specific Tregs in doses sufficient for clinical translation has been hampered by the low frequency of these cells in circulation. We previously demonstrated increased TCR β-chain repertoire diversity in CB Tregs (32). In this study, we expand on those bulk sequences to investigate paired gene expression and TCR profiles within the 6 identified APB and CB Treg clusters (Figure 2). We show APB Tregs to demonstrate increased clonal expansion as compared to CB Treg. We were able to define by gene expression the most expanded cluster in APB (C03), which expressed genes indicative of an activated memory phenotype (*S100A4*, *DUSP4*, *S100A10*, *LGALS1*, *LGALS3*). In contrast, the clusters that were moderately expanded in CB represent activated, functional Tregs expressing markers conferring adhesive and migratory potential, namely C01 (*JUNB*, *DUSP2*, and *ITGB1*), C02 (*IDI1*, *FCRL3*, *ID3*), and C06. Importantly, while C06 was shown to function as a contaminant in APB, increased co-expression of the cluster-defining genes (*CCL5*, *GZMK, CXCR3, LYAR*, and NKG7) with *FOXP3* and *IKZF2* in CB compared to APB suggests C06 CB Tregs to retain a regulatory phenotype more so than C06 in APB Tregs (Figure 2). We also show an increased number of CB TCRs map to putatively annotated autoreactive sequences, relative to TCRs from APB. Hence, polyclonal CB Tregs might provide a more comprehensive repertoire from which to seed the periphery. Indeed, a recent study has demonstrated the capacity to expand proinsulin (PI)-reactive Tregs from CB with increased yield compared to peripheral blood from subjects with T1D (137). The resultant pool of PI-specific Tregs was found to maintain lineage stability and suppressive function. These results coupled with our data support the notion that CB Tregs represent a population with increased phenotypic homogeneity alongside increased receptor diversity compared to APB Treg, thus serving as an ideal candidate for ACT in autoimmune diseases. Additionally, the potential to generate TCR redirected or chimeric antigen receptor (CAR) Treg has become a topic of great interest in the immunotherapy space (138–140). Our data suggest that the phenotypic stability and homogeneity of CB Tregs relative to APB might ameliorate concerns over lineage stability with TCR or CAR-directed Treg therapies.

We next examined the bulk transcriptomic profiles of CB and APB Tconv and Treg subsets after a 14-day expansion period (Figure 4). As expected, APB Tregs displayed increased expression of immunoregulatory markers (*FOXP3*, *IKZF2*, *TIGIT*, *TNFRSF9*) as compared to APB Tconv. In APB Tconv, we observed upregulation of *GZMA*, *GZMB*, *GNLY*, *IL7R*, and *IL18RAP*, which promote pro-inflammatory signaling (141–143) and have been associated with reduced suppressive capacity (144) as well as autoimmune susceptibility (145). In fact, a number of these genes have been implicated in the progression of autoimmune diseases, including T1D (146), hence avoiding cellular contaminants that express them is paramount to the development of an effective therapy with low risk of exacerbating the underlying pathology. While CB Tregs upregulate genes that promote regulatory function relative to CB Tconv (*LGALS3*, *LGMN*, and *HES1*), our data also support a more immunoregulatory phenotype in CB Tconv versus APB Tconv, as evidenced by upregulation of *IKZF2*, *SOX4*, *TIGIT*, and *TNFRSF9*. This observation suggests that, even in naïve CD4^+^ Tconv subsets, the default developmental program during the perinatal period may preferentially induce a regulatory gene expression profile. Furthermore, we observed increased expression of *GBP1* and *STAT1* by APB Tregs as compared to CB Tregs, likely signifying a more pro-inflammatory phenotype (147) and potentially, reduced suppressive capacity (148). In contrast, CB Tregs were enriched in markers that promote homing to the gut and activation and migratory potential (*GPR55*, *DST*) as well as stem-cell and recent thymic emigrant phenotypes (*TCF4*, *THEMIS*). Interestingly, single nucleotide polymorphisms (SNPs) within the chromosome region containing *THEMIS* have recently been associated with younger age at T1D diagnosis (149), suggesting that alterations in this gene may contribute to aberrant thymocyte selection and thereby, autoimmunity. Collectively, our data imply that expanded CB Tregs may better maintain their ability to traffic to sites of inflammation without the acquisition of an ex-Treg phenotype observed to be enriched in APB Tregs in T1D (28).

In examining Treg surface phenotype, we showed both APB and CB Tregs to express more markers of Treg activation/suppression than Tconv, namely TIGIT, HLA-DR, and CD28. Indeed, TIGIT^+^ Tregs have been shown to be more activated, to express early activation molecules such as CD69 and checkpoint molecules such as PD-1, and to suppress CD8 T cell and NK cell responses (150). Similarly, HLA-DR^+^ Tregs have been identified as a highly suppressive population, which is depleted in patients with acute post-transplant rejection (151). PD-1 expression was reduced amongst both APB and CB Tregs as compared to Tconv. While PD-1 expression has been shown to facilitate Treg activation and suppression of Teff responses through interaction with PD-L1 (152), high levels of PD-1 expression have been associated with T cell exhaustion (153). Indeed, Tregs expressing high levels of PD-1 have been shown to exhibit functional impairments, such as reduced suppressive capacity and increased IFNγ secretion (154). We recapitulated our previous observations in Figure 4 that show CB Tconv exhibit an immunoregulatory phenotype, with increased TIGIT as compared to APB Tconv. Finally, increased CD226 expression concomitant TIGIT in CB Tregs indicates increased activation, a finding consistent with increased cell yield following expansion cultures (32), while increased CD73 and CD95L expression is indicative of a more broadly suppressive population.

Our data suggest altered transcriptional profiles and suppressive properties of CB Treg relative to APB; however, we acknowledge a number of limitations in our current study. First, our analyses include comparisons across different donors due to longitudinal sample limitations and availability. It is possible that some of the observed differences in gene expression and phenotype are driven by genetic background of donors, and not a function of CB versus APB Tregs. We also acknowledge that some of our observations could relate to the relative imbalance in naïve and memory Treg subsets in CB and APB. Despite these limitations, we expect that the epigenetic profile of CB and APB may differ dramatically, even among the naïve Treg subset. Thus, studies are ongoing to examine the single cell differential chromatin accessibility profiles of CB and APB Tregs through the single cell Assay for Transposase Accessible Chromatin with sequencing (scATAC-seq). These data may also help to uncover the molecular basis for the more regulatory phenotype we observed in CB Tconv.

Our data suggest that modifications of the standard Treg sorting protocol to exclude cells expressing the surface marker CXCR3 present in our APB “contaminant” population as well as targeting/suppressing genes encoding pro-inflammatory markers and transcription factors (*BHLHE40*, *GBP1*) may result in a purer Treg population for use in ACT. Cumulatively, our observations suggest that APB-derived Tregs contain subsets of more differentiated and expanded Tregs as well as contaminants capable of producing cytotoxic molecules and proinflammatory cytokines, which could compromise the success of Treg ACT. In contrast, the CB Treg transcriptomic profile was more homogenous, supporting an undifferentiated regulatory phenotype, and reflects a predisposition for increased cell cycling and proliferation. In sum, clinical CB biobanks may serve as an important source material as the field continues to explore advanced cellular therapies, including the potential for highly specialized and gene and receptor edited cell products to induce durable immune tolerance.

## Data Availability Statement

The datasets generated for this study can be found in the GEO repository, with the accession numbers GSE137301 and GSE147794.

## Author Contributions

KM and LP researched and analyzed the data and wrote the manuscript. WV, AE, HS, and ML researched and analyzed the data and reviewed/edited the manuscript. HB analyzed the data and contributed to discussion. AP reviewed/edited the manuscript and contributed to discussion. MB and DP researched the data, contributed to discussion, and reviewed/edited the manuscript. RB analyzed the data and reviewed/edited the manuscript. JL reviewed/edited the manuscript. MH conceived of the study and reviewed/edited the manuscript. TB conceived of the study and wrote the manuscript.

## Conflict of Interest

The authors declare that the research was conducted in the absence of any commercial or financial relationships that could be construed as a potential conflict of interest.

## Footnotes

1 https://support.10xgenomics.com/single-cell-gene-expression/software/release-notes/build

2 https://support.10xgenomics.com/single-cell-gene-expression/software/pipelines/latest/algorithms/overview
