# Supplementary material for: Human Regulatory T Cells From Umbilical Cord Blood Display Increased Repertoire Diversity and Lineage Stability Relative to Adult Peripheral Blood
Source: Front Immunol. 2020 Apr 15;11:611. doi: 10.3389/fimmu.2020.00611 (PMC7174770; doi:10.3389/fimmu.2020.00611)
Supplement: Supplementary file 1 [file Data_Sheet_1.PDF]

Supplemental Materials for Human Regulatory T Cells from Umbilical Cord Blood Display Increased Repertoire Diversity and Lineage Stability Relative to Adult Peripheral Blood

March 02, 2020

Supplemental Figures:

See figures below.

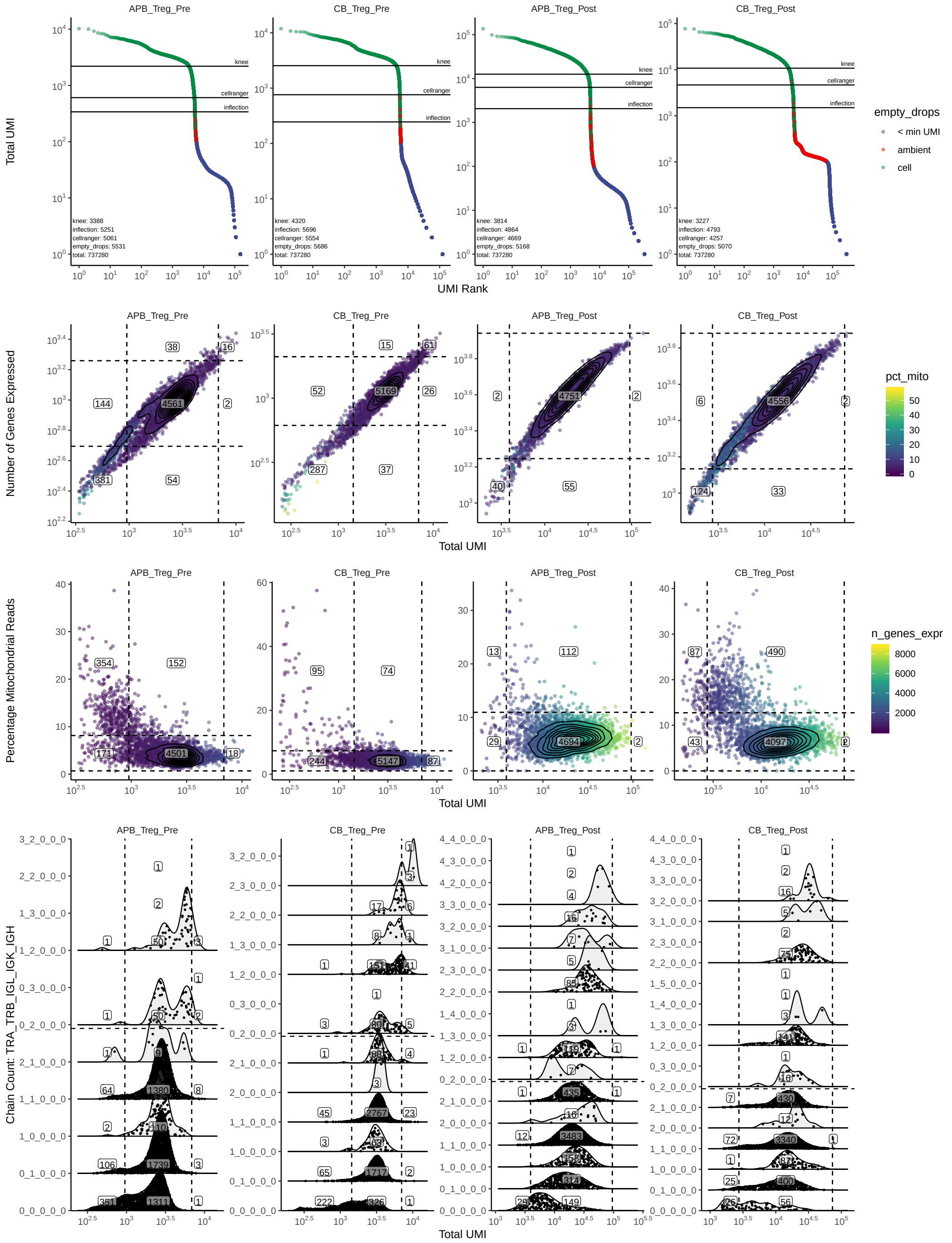

Supplemental Figure 1: Quality control of single-cell RNA-seq data. A.) Identification of barcodes containing intact cells versus ambient RNA. For each sample, the UMI Rank versus total number of UMI reads for each barcode is plotted. Each point is colored based on the result of the emptyDrops algorithm, and a line is drawn for common threshold-based filters. Barcodes identified as a cell using emptyDrops and above the inflection point were kept. B.) Joint distribution of total UMI reads and number of genes expressed, and colored by percentage of mitochondrial reads. Thresholds were determined for each marginal distributions based on 3 MAD above or below the median for total UMI and number of genes expressed. C.) Joint distribution of total UMI reads and percentage of mitochondrial reads, and colored by number of genes expressed. Thresholds were determined for each marginal distributions based on 3 MAD above or below the median for total UMI 3 MAD above for the percentage of mitochondrial reads. D.) Number of unique TRA, TRB, IGL, IGK, and IGH chains per barcode versus total number of UMIs and number of genes expressed. Below the horizontal dashed line are cells with 0-1 unique TRB chains and 0-2 unique TRA chains and are kept in this filter, and above the horizontal line are all other barcodes which are removed in this filter.

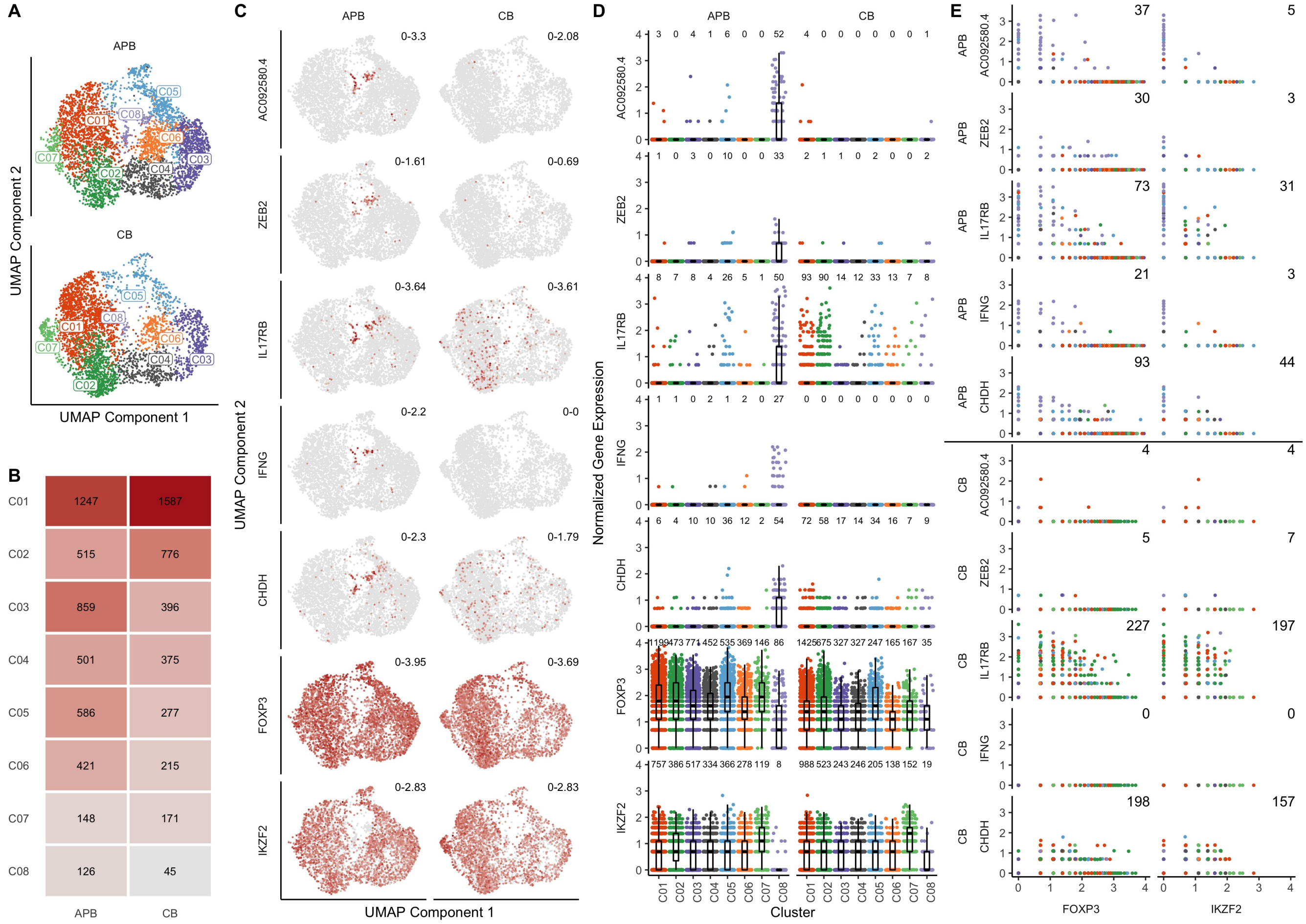

Supplemental Figure 2: Single cell gene expression profiles of expanded APB Tregs and expanded CB Tregs show the presence of a TH1/TH17-like contaminant cluster in APB. (A) UMAP plots for APB Tregs (n=1, top) and CB Tregs (n=1, bottom) colored by the assigned cluster number (C01-C08) shows the presence of a subpopulation (C08) that is more isolated from the rest and is more prevalent in APB. (B) Absolute number of cells belonging to each cluster and relative abundances for each cluster within expanded APB Tregs (left) and expanded CB Tregs (right) show differences in cluster composition. Notably, C08 was comprised of 126 cells (3 percent) of APB Tregs versus 45 cells (1 percent) of CB Tregs. (C) For APB Tregs (left) and CB Tregs (right), UMAP plots are colored by expression of the five most differentially expressed genes in C06 (AC092580.4, ZEB2, IL17RB, IFNG, and CHDH) as well as two canonical Treg genes (FOXP3 and IKZF2). (D) Expression of AC092580.4, ZEB2, IL17RB, IFNG, and CHDH, FOXP3 and IKZF2 in each cluster shows marked increases in "contaminant" gene expression in C08 and lower FOXP3 and little IKZF2 expression in Tregs (left) and CB Tregs (right). (E) For all clusters, pairwise scatterplots between canonical Treg genes FOXP3 (left) and IKZF2 (right) and the identified "contaminant" genes (AC092580.4, ZEB2, IL17RB, IFNG, and CHDH) shows increased co-expression in CB Tregs (lower) compared to APB Tregs (upper). (D-E) For each data point, the color corresponds to the cluster number as presented in panel A.

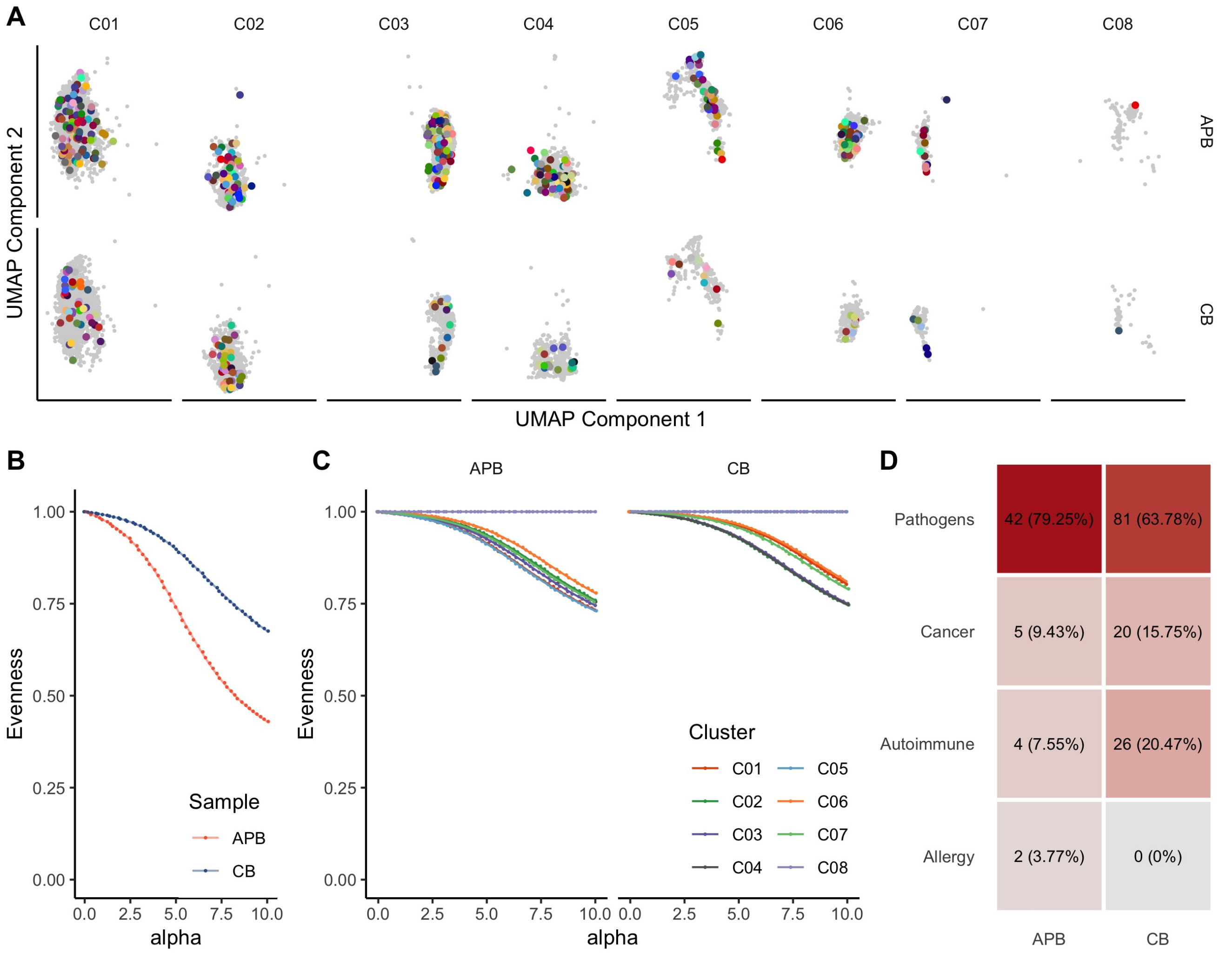

Supplemental Figure 3: T cell receptor (TCR) profiling from scRNA-seq data identifies clonally expanded subpopulations post-expansion. (A) UMAP plot colored by clonotypes with single occurrences (gray) and multiple occurrences (each clonotype distinctly colored) identifies clusters with expansion (UMAP Components 1 and 2 are the same as those presented in Figure 2). (B) Alpha value (i.e., clonotype frequency) versus evenness value (42) for APB Tregs (orange) and CB Tregs (blue) shows that along the range of the frequency distribution, APB Tregs show lower evenness at all alpha levels and thus, greater clonal expansion than CB Tregs. (C) When APB Tregs (n=1, left) and CB Tregs (n=1, right) are split by cluster (C01-C08, as defined in the last figure), all clusters but APB C08, CB C05, and CB C08 are expanded. (D) Putative antigen specificity composition of TCRs in each sample show an increased number of clones mapping to known reactivities, including autoimmune clones, in CB Tregs (right) compared to APB Tregs (left).

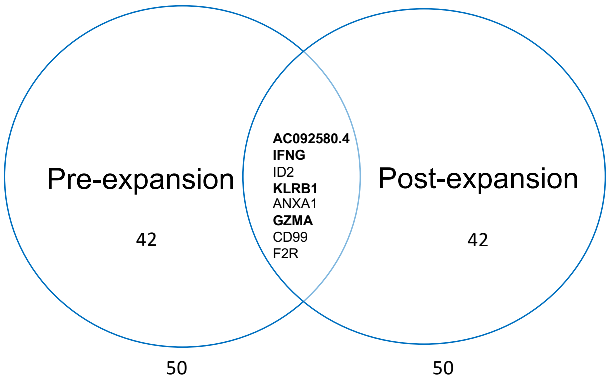

Supplemental Figure 4: Venn diagram depicting the overlap of the top 50 (ranked on p-value) differentially expressed genes in the contaminant cluster for pre-expansion (C06) and post-expansion (C08) data. Bold text indicate those genes highlighted in the text that support similar contaminant phenotypes.

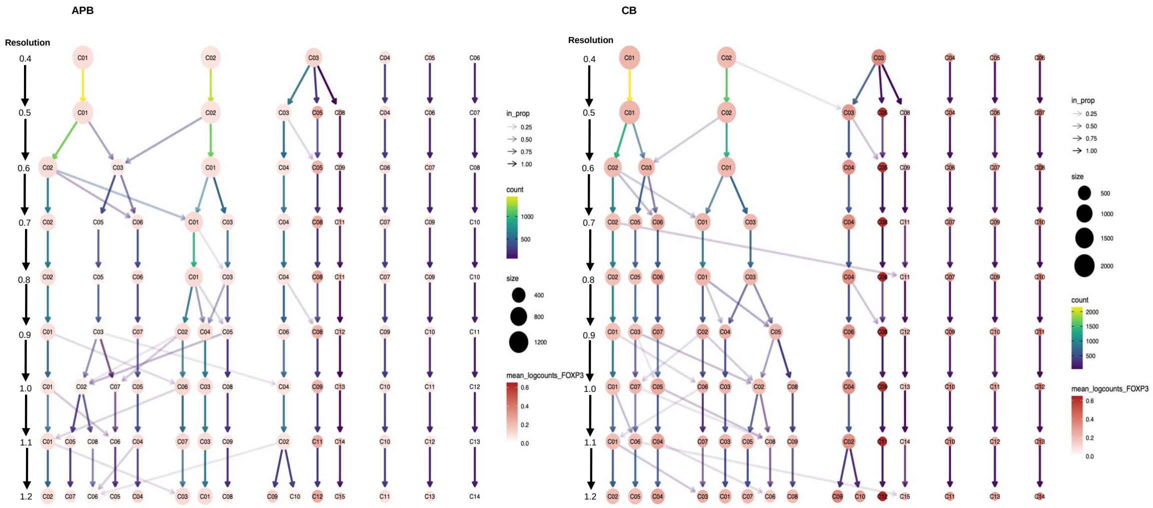

Supplemental Figure 5: Clustree showing the robustness of the contaminant cluster in pre-expansion data (C06) to cluster resolution. Each row is increasing resolution as indicated on the left side. Each node is colored by mean FOXP3 expression and sized according to cluster size. The opacity of edges is based on the proportion of cells conserved between the two nodes it connects, and colored based on the absolute number of cells conserved.

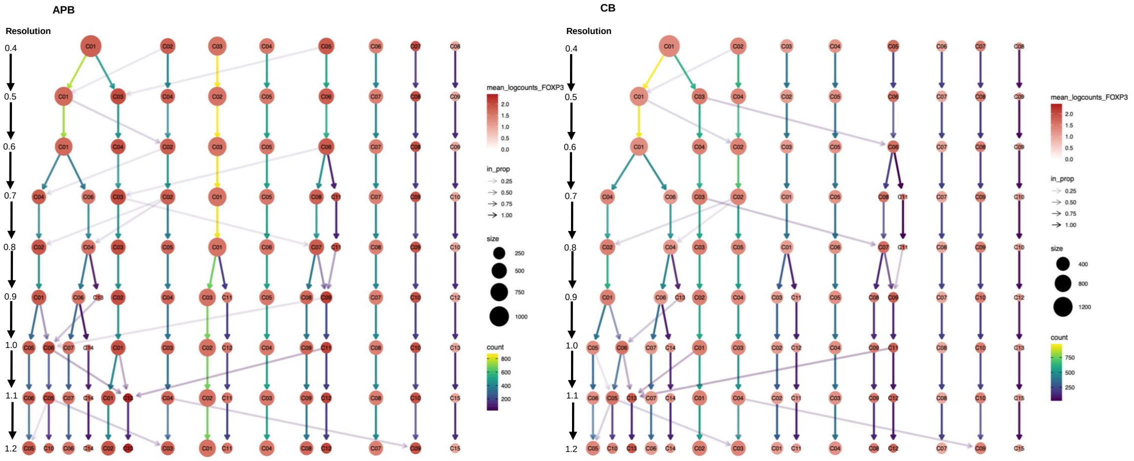

Supplemental Figure 6: Clustree showing the robustness of the contaminant cluster in post-expansion data (C08) to cluster resolution. Figure is otherwise identical to the previous one.

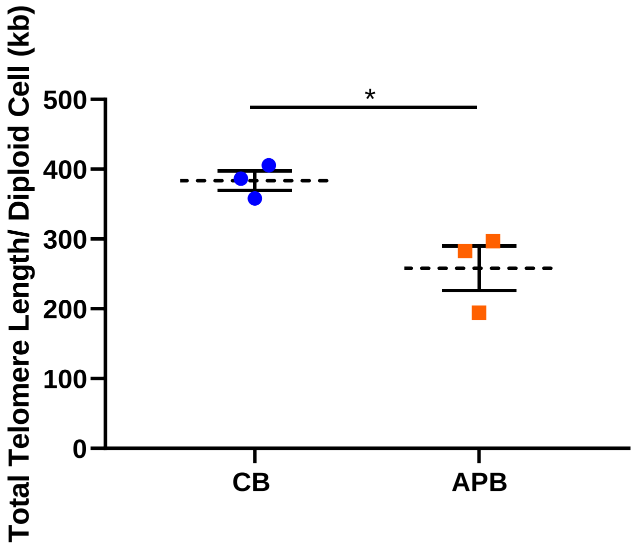

Supplemental Figure 7: Differences in absolute telomere length between post-expansion CB and APB Tregs. Absolute telomere length was assessed via qPCR. We observed CB Treg (n=3) to retain longer telomeres post-expansion than APB Treg (n=3). Data were analyzed in GraphPad PRISM v8. Statistics are the result of an unpaired Welch's T Test with two-tailed *$p<0.05$.

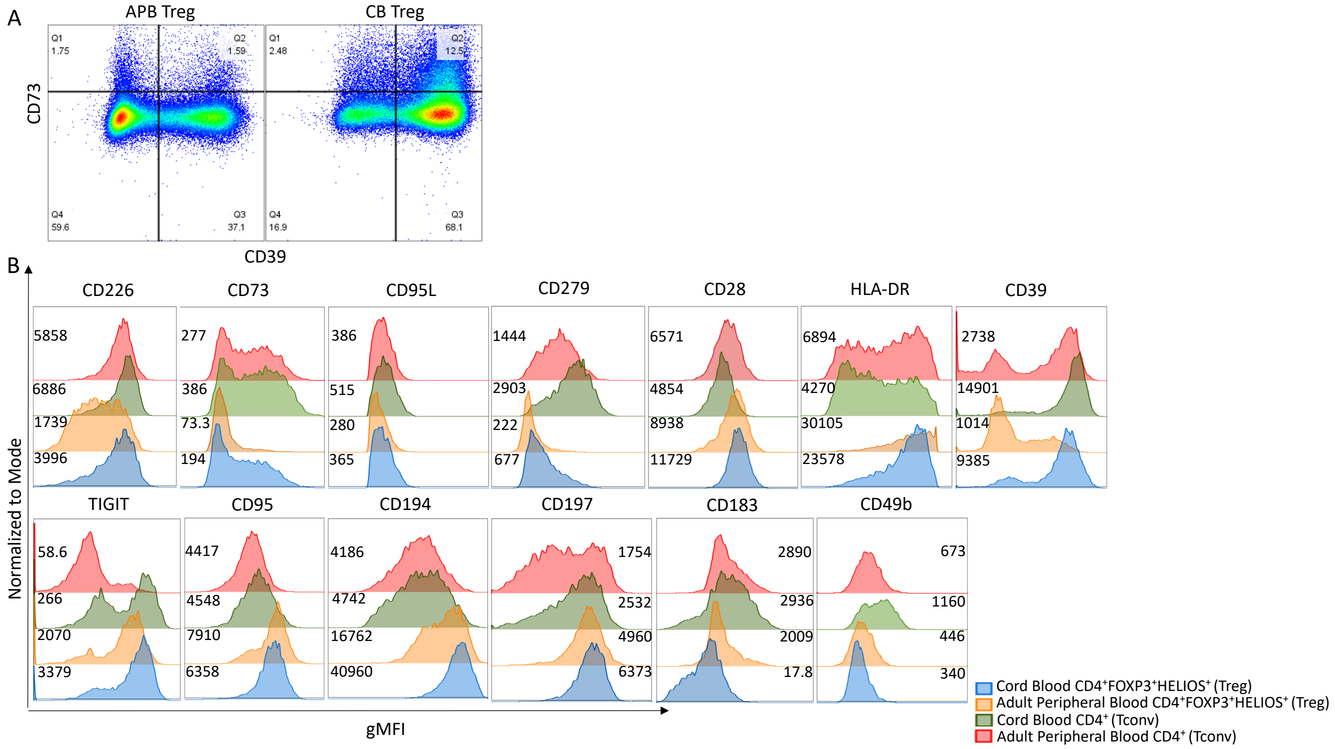

Supplemental Figure 8: Figure S11: Flow cytometric analysis shows increased activation and suppressive phenotype of CB Tregs compared to APB Tregs. A) Representative FACS plots of expanded APB and CB Tregs post 48 hr a-CD3/28 re-stimulation shows an increased number of CB Tregs to co-express ecto-enzymes CD39 and CD73 as compared to APB Tregs. B) Representative histograms of expression of markers displayed in Figure 6 overlayed for CB Tregs, APB Tregs, CB Tconv, and APB Tconv. Values displayed are gMFI of the marker on the whole subset (Treg or Tconv).

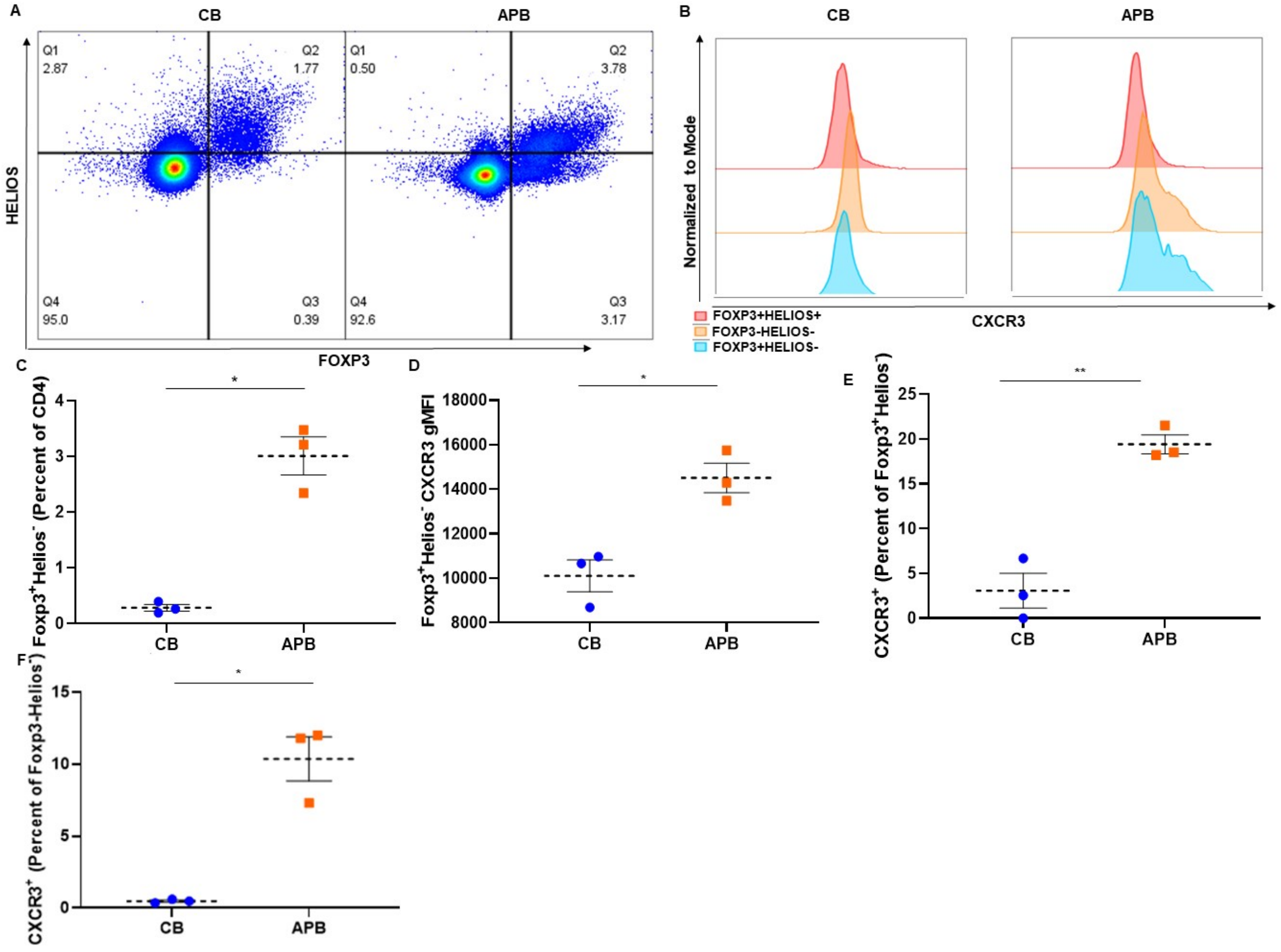

Supplemental Figure 9: APB contains more CXCR3 expressing non-tTreg contaminants as compared to UCB. A) Representative FACS plots of Foxp3 and Helios expression within CBMC and PBMC B) Representative histograms of CXCR3 expression across T cell subsets within PBMC and CBMC, with Foxp3+Helios- and Foxp3-Helios- subsets displaying greater expression than Foxp3+Helios+ Treg. C) Foxp3+Helios- T cells are increased in APB PBMC compared to CBMC. D) The Foxp3+Helios- subset expresses more CXCR3 in APB PBMC than CBMC, E) posesses more CXCR3+ cells, and F) The Foxp3-Helios- subset is also comprised of an increased number of CXCR3+ cells in APB as compared to CB. **$p<0.01$, *$p<0.05$ by Welch's T test

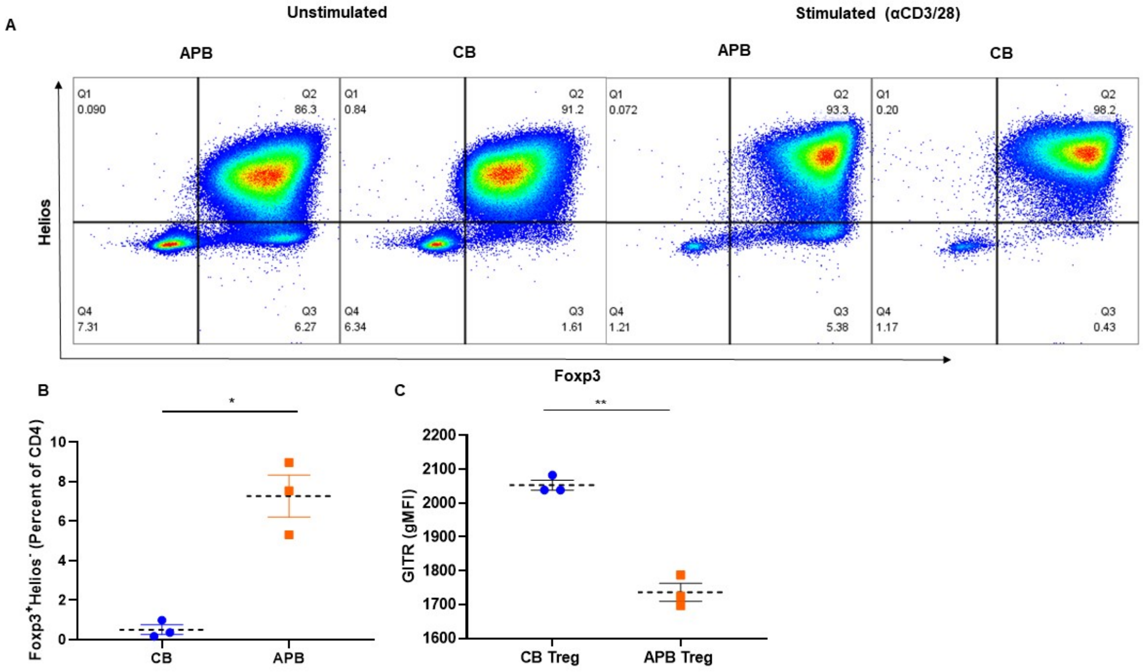

Supplemental Figure 10: Expanded APB Tregs contain more non-tTreg contaminants and lack co-stimulatory marker expression as compared to expanded UCB Tregs. A) Representative FACS plots of expanded APB and CB Tregs pre and post-aCD3/28 stimulation. B) APB Treg contain more Foxp3+Helios- contaminants than CB Treg post expansion, and C) CB Treg express increased GITR as compared to APB Treg. $^{**}p<0.01$, $^{*}p<0.05$ by Welch's T test

Supplemental Tables:

Supplemental Table 1: Antibodies used for flow cytometry panels. A comprehensive list of the antibodies used to analyze phenotypic differences between CB and APB.

Supplemental Table 2: Differentially expressed genes per scRNAseq cluster for pre-expansion dataset. A table of the top 50 differentially expressed genes per cluster by p value.

Supplemental Table 3: Differentially expressed genes per scRNAseq cluster for post-expansion dataset. A table of the top 50 differentially expressed genes per cluster by p value.

Supplemental Table 4: Differentially expressed genes from microarray analysis. A table of the top 100 differentially expressed genes per comparison ranked by p value and by log2FC of 1.5.
